# Infection of neonates with *Staphylococcus aureus* and methicillin-resistant *Staphylococcus aureus* at Dormaa Presbyterian Hospital, Ghana

**DOI:** 10.1128/spectrum.01749-24

**Published:** 2025-05-15

**Authors:** Jerome Adinkrah Obeng, William Gariba Akanwariwiak, Eugene Adade, Augustina Angelina Sylverken

**Affiliations:** 1 Department of Theoretical and Applied Biology, Kwame Nkrumah University of Science and Technology 98763, Kumasi, Ghana; University of Pretoria, Pretoria, South Africa

**Keywords:** *Staphylococcus aureus*, MRSA isolates, MRSA infection, neonatal intensive care unit

## Abstract

*Staphylococcus aureus* is the second most common pathogen found in all cases of nosocomial infections globally. Methicillin-resistant *Staphylococcus aureus* (MRSA) is a common cause of infection among preterm and critically ill newborns in neonatal intensive care units. Newborns are predominantly susceptible to *S. aureus* and MRSA colonization and infection due to their weaker immune systems. Treating infections in newborns associated with *S. aureus* and MRSA has proven challenging due to decreasing susceptibility to first-line antibiotics, leaving clinicians with few treatment options. This cross-sectional study aimed to determine the prevalence of hospital-acquired MRSA among neonates at the Dormaa Presbyterian Hospital in Ghana, the susceptibility profile to selected antibiotics, and the associated risk factors. Venous blood samples were taken from each of the neonatal participants and inoculated into Brain Heart Infusion broth. Standard biochemical tests were performed, and 36.0% (9/25) of the *S. aureus* isolates were identified as MRSA. The MRSA isolates were more susceptible to ciprofloxacin, levofloxacin, gentamicin, co-trimoxazole, tetracycline, and cephalexin but resistant to cloxacillin, ampicillin, roxithromycin, and lincomycin. The study found that gestational period (χ^2^ = 3.865, *P* = 0.049) and longer length of hospital stay (χ^2^ = 10.911, *P* = 0.012) were statistically significant for *S. aureus* and MRSA infection. Surveillance systems should be put in place by health authorities at the hospital, targeting decolonization of MRSA strains at the hospital as well as monitoring antibiotic resistance that occurs through inappropriate access and use of antibiotics.

**IMPORTANCE:**

The methicillin-resistant *Staphylococcus aureus* (MRSA) threat has become a source of concern for medical facilities and patients worldwide. MRSA infections are more difficult to treat, resulting in higher rates of morbidity and mortality. Prematurity, low birth weight, method of delivery, mode of resuscitation after birth, feeding method, prolonged hospital stay, kangaroo mother care, and overcrowding in hospitals are all risk factors for MRSA infection. Overcrowding is a common issue in the Dormaa Presbyterian Hospital (DPH), as it is in most hospitals around the country. The study intended to bring to the notice of mothers, hospital staff, and caregivers the risk factors associated with *Staphylococcus aureus* and methicillin-resistant *Staphylococcus aureus* among neonates at DPH.

## OBSERVATION

*Staphylococcus aureus*, a member of the family *Staphylococcaceae,* is a gram-positive and coagulase-positive bacterium, which is usually a commensal member of the human microbiota. However, it can potentially develop into an infectious agent due to its ability to adapt to various hosts and environmental conditions and cause a variety of infections (1). Such infections affect the skin, soft tissues, and lower respiratory tract. *S. aureus* also causes infections associated with medical equipment and potentially serious deep-seated infections such as osteomyelitis and endocarditis (2). *S. aureus* develops antibiotic resistance, particularly methicillin resistance, as a result of the overuse of methicillin. The bacterium has the mecA gene, which helps it to withstand the effects of methicillin and beta-lactam antibiotics (3, 4). Twenty years after the first methicillin-resistant *Staphylococcus aureus* (MRSA) case was reported, the first infant MRSA infection case that emerged in a neonatal intensive care unit (NICU) was reported (5). MRSA has since turned out to be a common cause of infections affecting premature and severely ill newborns in NICUs (6). Treating MRSA has proved to be difficult, resulting in morbidity and mortality, as well as longer hospital stays. This imposes a substantial financial burden on patients and the scarce healthcare resources in the management of the infection (7, 8). Prematurity and younger gestational age, low birth weight, feeding methods, prolonged stay in the hospital, kangaroo mother care, and the presence of eye mucus in newborns’ eyes are risk factors for MRSA infection in newborns (9, 10).

Methicillin resistance in *Staphylococcus aureus* has been found to be higher than 20% in all World Health Organization (WHO) regions, and as high as 80% in some WHO member countries (11). MRSA rates in 32 African countries range between 12% and 80%, with some countries exceeding 82%, according to the Africa CDC (12, 13). In Uganda, for example, high prevalence rates of 31.5%– 42.0% have been recorded among patients and healthcare staff (12, 14). In Ghana, data on MRSA are scanty; however, the few available data indicate that MRSA rates range from 21% to over 75% in the general population (15–18). Although there has been a review of MRSA infection in neonates, children, and adolescents in Ghana (19), no empirical study on neonates has been conducted in healthcare settings in Ghana. As such, this study sought to determine the prevalence of *S. aureus* and MRSA, their antibiotic susceptibility profile, and the associated risk factors among neonates at Dormaa Presbyterian Hospital (DPH) in the Bono region of Ghana.

The study was conducted at Dormaa Presbyterian Hospital’s Neonatal Unit in the Bono Region of Ghana. The hospital, located in the Dormaa Central municipality, receives referrals from Dormaa East, Dormaa West, Jaman South, and the nearby country Ivory Coast. The Neonatal unit ward is divided into Neonatal Intensive Care Unit One (NICU 1) and Neonatal Intensive Care Unit Two (NICU 2). NICU 1 admits newborn babies from day zero (0) to day seven (7), while NICU 2 admits babies from day seven to 8 weeks. These neonates might have been delivered at the hospital or referred from health facilities, as well as traditional birth attendants (TBAs), within the hospital’s catchment areas.

This cross-sectional study recruited 110 neonates on admission at NICU 1 and NICU 2 of Dormaa Presbyterian Hospital, along with their index mothers, using purposive sampling. The inclusion criteria included documented parental informed consent for the study participants. Newborns admitted to NICU 1 and NICU 2 who showed signs of neonatal sepsis were recruited. Similarly, neonates who had been hospitalized for at least 48 hours were also included in the study. Neonates excluded were those undergoing antibiotic therapy, those who had spent less than 24 hours on admission, and those who expired before sample collection commenced.

One milliliter (1 mL) of venous blood was taken from each neonate and inoculated into a blood culture bottle containing 10 mL of Brain Heart Infusion broth (HiMedia, India). The collected samples were immediately sent to the Microbiology Unit of the hospital’s laboratory for further analysis. With the help of the Baby Assessment Form completed by doctors and midwives immediately after delivery, a questionnaire was administered to each participant, capturing biodata such as age (in days) and gender. Similarly, a structured questionnaire was completed on each of the neonates addressing the duration of admission, gestational age, birth, and weight. In addition, information about appearance, pulse, grimace, activity, and respiration score (APGAR score) in the first and fifth minutes after birth was recorded. Additionally, information on the mode of breastfeeding was captured. Information on baby resuscitation after birth was also recorded to help identify some neonatal risk factors.

Blood samples collected were incubated at 37°C for 18–24 hours. After the incubation, each sample was aseptically cultured on mannitol salt agar (MSA) (Oxoid, UK) at 37°C for 18–24 hours. Hale-yellow colonies on MSA after incubation were suggestive of *S. aureus*. Plates without growth for the first 18–24 hours were incubated for another 24 hours and were discarded if there was no growth. Single isolated colonies were picked from plates with significant counts and subcultured on MSA (Oxoid, UK) to obtain pure cultures. Based on morphological characteristics, *S. aureus* was identified by the presence of golden yellow colonies with a round, convex, opaque, and smooth-glistening surface with a diameter of about 2–3 mm (20).

MRSA was detected using 30 µg cefoxitin on Mueller-Hinton agar (MHA) (Oxoid, UK). A suspension of the isolate was prepared with normal saline to obtain a 0.5 McFarland suspension. About 25 mL of the suspension was inoculated on the MHA and allowed to dry. Thirty micrograms of cefoxitin was carefully applied on the surface of the medium and incubated at 37°C for 18–24 hours. If the inhibition zone for cefoxitin was ≤21 mm, the culture was considered positive for MRSA. The result was reported as methicillin-susceptible *S. aureus* (MSSA) or MRSA based on the zone of inhibition on cefoxitin by comparing them to the Clinical and Laboratory Standards Institute (CLSI) zone measurement chart (21).

The Kirby-Bauer disc diffusion method on MHA (Oxoid, UK) was used to test the antibiotic sensitivity of all the isolates, as recommended by the CLSI (21). The antibiotics used for AST were ampicillin (20 mcg), cephalexin (30 mcg), ciprofloxacin (5 mcg), co-trimoxazole (25 mcg), cloxacillin (5 mcg), gentamicin (10 mcg), levofloxacin (5 mcg), lincomycin (2 mcg), linezolid (30 mcg), roxithromycin (15 mcg), and tetracycline (30 mcg) all from Oxoid, UK. These antibiotics were carefully placed on the surface of the media using sterile forceps. The plates were inverted and incubated at 37°C for about 18–24 hours. Using a caliper and a ruler, inhibition zones around the discs were measured. Finally, the results were recorded and interpreted as resistant (R), intermediate (I), and sensitive (S) by comparing them to the CLSI zone measurement chart.

Data from the laboratory analysis and questionnaires were entered into Microsoft Office Excel 2019. Categorical variables were presented as numbers and proportions. Chi-square analysis was used to determine associations between categorical variables. Each categorical variable was considered significant if its *P*-value was ≤0.05. All analyses were performed using Statistical Package for the Social Sciences version 26, USA.

In total, 110 infants were included in the study. The socio-demographic characteristics of neonates included in the study indicate that the majority were males, representing 59.1% (65/110). The majority of newborns, representing 36.4% (40/110), were 4–5 days old. A total of 67.0% (74/110) of the neonates were delivered at the hospital where the study was conducted, while 7.0% (8/110) were delivered by TBAs and referred to the hospital’s NICU for good health care after delivery, as shown in Table 1

**TABLE 1:** Demographic characteristics of neonatal participants

Variable	Category	Frequency	Percentage
Gender	Male	65	59.1
	Female	45	40.9
Age (days)	2–3	18	16.4
	4–5	40	36.4
	6–7	25	22.7
	>7	27	24.5
Place of delivery	Hospital delivery	74	67.0
	Health facility referral	28	26.0
	TBA delivery	08	7.0

The prevalence of *S. aureus* was 22.7% (25/110), while non*-S. aureus* growth was 11.8% (13/110). Of the total 25 positives, *S. aureus* was found in 56.0% (14/25) of males and 44.0% (11/25) of females. There was no culture growth of blood samples in 65.5% (72/110) of the study participants.

In terms of prevalence based on places of birth, hospital delivery recorded the highest prevalence of 52.0% (13/25), followed by health facility referral with 36.0% (9/25), while TBA recorded the least with 12.0% (3/25).

The gestational period of 37–42 weeks recorded the highest neonates of 80.9% (89/110). Periods <37 weeks (preterm) had the least neonatal participants at 19.1% (21/110). Most neonatal participants weighed between 2.6 and 3.5 kg after birth, accounting for 58.2% (64/110), while only 0.9% (1/110) weighed below 1.5 kg after birth. Fifty-one percent (23/45) of the neonates underwent bagging and masking form of resuscitation to enhance their breathing, while 15.6% (7/45) were put on ventilator support after delivery. Also, 36.4% (40/110) of the neonates had spent between 2 and 3 days on admission, while 17.2% (19/110) spent >7 days on admission. Majority of the neonates representing 61.8% (68/110) were fed with breast milk through sucking, while expressed breast milk (EMB) was used to feed 20% (22/110) of the neonates. All information on neonatal factors is shown in Table 2.

**TABLE 2:** Neonatal factors

Variable	Category	Frequency	Percentage
Gestational period	<37 weeks (preterm)	21	19.1
	37–42 weeks	89	80.9
Birth weight	<1.5 kg	1	0.9
	1.5–2.5 kg	29	26.4
	2.6–3.5 kg	64	58.2
	>3.5 kg	16	14.5
Resuscitation during and after birth	Yes	45	40.9
	No	65	59.1
Type of resuscitation	Suction	15	33.3
	Bag-valve-mask	23	51.1
	Ventilator support	7	15.6
APGAR score in first minute	<7	37	33.6
	>7	73	66.4
APGAR score in fifth minute	<7	3	2.7
	>7	107	97.3
Period of admission	0–1 day	1	0.9
	2–3 days	40	36.4
	4–5 days	32	29.1
	5–6 days	18	16.4
	>7 days	19	17.2

High phenotypic resistance of MRSA to the antibiotics tested was recorded for cloxacillin 88.9% (8/9) followed by ampicillin 55.6% (5/9), while roxithromycin and lincomycin recorded 44.4% (4/9) resistance each. High sensitivity of MRSA to the antibiotics tested was recorded for ciprofloxacin 88.9% (8/9), levofloxacin 88.9% (8/9), and gentamicin 88.9% (8/9) as shown in Table 3.

**TABLE 3:** Antimicrobial susceptibility distribution profile of MRSA isolates

Antibiotic	Resistant		Sensitive		Intermediate	
	No.	%	No.	%	No.	%
Ampicillin	5	55.6	2	22.2	2	22.2
Co-trimoxazole	3	33.3	6	66.7	0	0
Cephalexin	3	33.3	5	55.6	1	11.1
Tetracycline	3	33.3	6	66.7	0	0
Ciprofloxacin	0	0	8	88.9	1	11.1
Levofloxacin	1	11.1	8	88.9	0	0
Linezolid	0	0	1	11.1	8	88.9
Cloxacillin	8	88.9	1	11.1	0	0
Roxithromycin	4	44.4	4	44.4	1	11.1
Lincomycin	4	44.4	2	22.4	3	33.3
Gentamicin	1	11.1	8	88.9	0	0

The chi-square test was used to determine whether there was an association between neonatal risk factors and methicillin response to *S. aureus*. Only two of the risk variables statistically showed a significant effect on methicillin response to *S. aureus* (*P* < 0.05). The association between gestational period and methicillin response to *S. aureus* was statistically significant (χ^2^ = 3.865, *P* = 0.049). The study also found a statistically significant association between the period of admission and methicillin response to *S. aureus* (χ^2^ = 10.911, *P* = 0.012) as shown in Table 4.

**TABLE 4:** Association between neonatal risk factors and methicillin response to *S. aureus*

Variable	Category	Methicillin response		Chi-square	*P* value
		MRSA ***N*** (%)	MSSA ***N*** (%)		
Gestational period	<37 weeks	2 (22.2)	0 (0.0)	3.865	**0.049^*a*^**
	37–42 weeks	7 (77.8)	16 (100.0)		
Birth weight	1.5–2.5 kg	3 (33.3)	1 (6.2)	4.384	0.112
	2.6–3.5 kg	6 (66.7)	12 (75.0)		
	>3.5 kg	0 (0.0)	3 (18.8)		
Resuscitation during and after birth	Yes	6 (66.7)	7 (43.8)	1.212	0.271
	No	3 (33.3)	9 (56.2)		
APGAR score in first minute	<7	5 (55.6)	5 (31.2)	1.418	0.234
	>7	4 (44.4)	11 (68.8)		
APGAR score in fifth minute	<7	1 (11.1)	0 (0.0)	1.852	0.174
	>7	8 (88.9)	16 (100.0)		
Period of admission	2–3 days	0 (0.0)	3 (18.8)	10.911	**0.012**
	4–5 days	2 (22.2)	7 (43.8)		
	5–6 days	1 (11.1)	5 (31.2)		
	7–8 days	6 (66.7)	1 (6.2)		
Mode of feeding with breast milk	Sucking	5 (55.6)	9 (56.2)	1.066	0.587
	EMB	1 (11.1)	4 (25.0)		
	Both sucking and EMB	3 (33.3)	3 (18.8)		

^*a*^ The two bolded figures were the risk factors found to be associated with MRSA infection at NICU when chi square test was performed.

The association between staphylococcal colonization and infection development is evident, particularly in neonates (22), since its emergence in the early 1960s (23). *S. aureus* methicillin resistance has been traced to the acquisition of the mecA gene. The mecA gene encodes an extra penicillin-binding protein (PBP2a), a peptidoglycan transpeptidase that aids in conferring resistance to all beta-lactam antibiotics used in treating MRSA infections (24).

The prevalence of *S. aureus* in this study was 22.7%. Compared with other studies elsewhere in Africa (25–28), it suggests that *S. aureus* may be endemic in Africa, particularly in western, central, and eastern regions. This may be due to poor hygiene practices as a result of the low socioeconomic status of these countries (12).

In this study, 64% of the *S. aureus* isolates were MSSA, while 36% were MRSA. The 36% MRSA recorded agrees with the 32% of MRSA in hospitalized neonates in a Danish NICU (29). This could be attributed to the fact that sampling (sample sizes) for both studies was nearly the same. In contrast to other studies that showed low MRSA infection rates of 3.9% and 4.4% (30, 31) in NICUs, our study identified a higher MRSA infection rate in the newborns. This could be due to geographical variation in the places of isolation, as well as overcrowding of the NICU in this study. Overcrowding has previously been identified as a risk factor for MRSA infection in neonates in Nigeria (32).

Ampicillin and roxithromycin resistance were lower, and this could be due to low or no exposure of the study participants to these drugs since birth. Nonetheless, resistance recorded in ampicillin and roxithromycin could be attributed to abuse, resulting from clinicians’ over-prescription without proper diagnosis and patients’ incomplete treatment courses (33).

High susceptibility was recorded among ciprofloxacin, levofloxacin, and gentamicin. The evidence of gentamicin is consistent with other studies reported (34–36). This could be due to the limited use of gentamicin in MRSA treatment among the study participants or no exposure of the study participants to gentamicin since the drug is not easily accessible and affordable. Notwithstanding if it could be accessed, they are normally in the form of intravenous fluids, which need to be administered through the vein by a qualified clinician. This creates some form of inconvenience for potential users.

The chi-square test was performed to determine the associations between neonatal risk factors and methicillin response to *S. aureus*. It was revealed that the gestational period was statistically significant (χ^2^ = 3.865, *P* = 0.049). Our findings corroborate a report by Khoury et al. (37) from a level III-IV NICU with 18 beds in Washington, USA, where the gestational period was found to be strongly associated (*P* = 0.003) with an increased risk of MRSA colonization and infection in newborns.

It is expected that health education given to pregnant women by health care practitioners during pregnancy would enhance mothers’ knowledge on how to prevent sepsis in newborns due to MRSA infection (38). However, due to non-adherence to the guidelines and education by these mothers, it predisposes the newborns to a greater risk of developing sepsis due to MRSA infection, as evident in a study (39). Conditions such as low birth weight (40), birth asphyxia (41), sepsis and jaundice (42), meconium-stained liquor (43), fetal macrosomia (44), and oligohydramnios (45) are common neonatal conditions that necessitate admission after birth. Maraqa et al. (46) found prolonged hospital stay (duration of admission) as a variable that was statistically associated with the risk of MRSA infection (46). This is consistent with our findings because we found the period of admission to be significant (χ^2^ = 10.911, *P* = 0.012) to *S. aureus* and MRSA infection. The reason could be due to health authorities in the hospital not adhering to strict protocols when it comes to infection prevention practices in high-risk areas such as the NICU in the hospital, as evident in a study in a NICU in Botucatu, Brazil (47).

Since the study focused solely on neonates admitted to the hospital’s NICU, the findings may lack generalizability to the total population of *S. aureus* and MRSA cases recorded in the hospital. Also, this study was unable to perform spa typing and sequence typing on *S. aureus* isolates, which would have provided information on the prevalence of spa and sequence types circulating at the NICU of DPH.

The study found *S. aureus* and MRSA to be prevalent among neonates at the NICU of Dormaa Presbyterian Hospital. High antibiotic resistance to cloxacillin, ampicillin, and roxithromycin was detected among MRSA isolates. The MRSA isolates were more susceptible to ciprofloxacin, gentamicin, and levofloxacin. Neonatal factors such as gestational period and length of hospital stay were possible independent risk factors for *S. aureus* and MRSA infection among the neonates. Surveillance systems should be implemented at the hospital to monitor antibiotic resistance. Regular sampling of frequently touched surfaces and hospital equipment around the neonates in the NICU should be analyzed for MRSA to ensure the unit is adequately cleaned to reduce colonization and transmission of MRSA. Ciprofloxacin, gentamicin, and levofloxacin could be considered as first-line treatment for sepsis in relation to MRSA infection at the NICU of the hospital.

## Data Availability

The data that support the findings of this study are fully displayed in this article.
